# 3,4,5-Trihy­droxy­benzohydrazidium perchlorate–3,4,5-trihy­droxy­benzohydrazide–water (1/1/1)

**DOI:** 10.1107/S1600536811030595

**Published:** 2011-08-02

**Authors:** Abeer A. Alhadi, Hamid Khaledi, Hapipah Mohd Ali

**Affiliations:** a Department of Chemistry, University of Malaya, 50603 Kuala Lumpur, Malaysia

## Abstract

The crystal studied of the title compound, C_7_H_9_N_2_O_4_^+^·ClO_4_^−^·C_7_H_8_N_2_O_4_·H_2_O, was found to be a racemic twin with a 0.72 (18):0.28 (18) domain ratio. The hydrazidium group is close to planar, with an r.m.s deviation of 0.105 Å; the hydrazide group deviates more from planarity, with an r.m.s deviation of 0.174 Å. In the crystal, the hydrazidium cation, hydrazide mol­ecule, perchlorate anions and water mol­ecules are linked through O—H⋯O, N—H⋯O and C—H⋯O hydrogen bonds into a three-dimensional supra­molecular network. In addition, the benzene rings of the hydrazidium and hydrazide units are connected *via* π–π inter­actions into infinite chains along the *c* axis; the centroid–centroid distances are 3.486 (3) and 3.559 (3) Å.

## Related literature

For the crystal structure of trimeth­oxy­benzohydrazidium chloride, see: Saeed *et al.* (2008 ▶) and of 3,4,5–trimeth­oxy­benzohydrazide hemihydrate, see: Zareef *et al.* (2006 ▶).
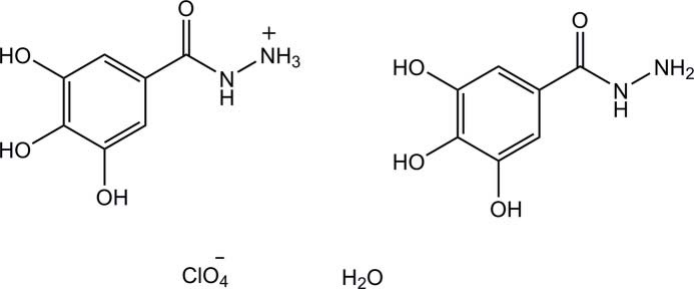


## Experimental

### 

#### Crystal data

C_7_H_9_N_2_O_4_^+^·ClO_4_^−^·C_7_H_8_N_2_O_4_·H_2_O
*M* *_r_* = 486.78
Orthorhombic, 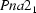

*a* = 20.1213 (7) Å
*b* = 12.9178 (4) Å
*c* = 7.0122 (2) Å
*V* = 1822.63 (10) Å^3^
*Z* = 4
Mo *K*α radiation
μ = 0.30 mm^−1^
*T* = 100 K
0.15 × 0.04 × 0.03 mm

#### Data collection

Bruker APEXII CCD diffractometer
Absorption correction: multi-scan (*SADABS*; Sheldrick, 1996 ▶) *T*_min_ = 0.957, *T*_max_ = 0.991
14383 measured reflections
3393 independent reflections
2653 reflections with *I* > 2σ(*I*)
*R*_int_ = 0.092

#### Refinement

*R*[*F* ^2^ > 2σ(*F* ^2^)] = 0.049
*wR*(*F* ^2^) = 0.109
*S* = 1.03
3393 reflections
335 parameters
22 restraints
H atoms treated by a mixture of independent and constrained refinement
Δρ_max_ = 0.39 e Å^−3^
Δρ_min_ = −0.53 e Å^−3^
Absolute structure: Flack (1983 ▶), 1545 Friedel pairs
Flack parameter: 0.28 (11)

### 

Data collection: *APEX2* (Bruker, 2007 ▶); cell refinement: *SAINT* (Bruker, 2007 ▶); data reduction: *SAINT*; program(s) used to solve structure: *SHELXS97* (Sheldrick, 2008 ▶); program(s) used to refine structure: *SHELXL97* (Sheldrick, 2008 ▶); molecular graphics: *X-SEED* (Barbour, 2001 ▶); software used to prepare material for publication: *SHELXL97* and *publCIF* (Westrip, 2010 ▶).

## Supplementary Material

Crystal structure: contains datablock(s) I, global. DOI: 10.1107/S1600536811030595/lx2194sup1.cif

Structure factors: contains datablock(s) I. DOI: 10.1107/S1600536811030595/lx2194Isup2.hkl

Supplementary material file. DOI: 10.1107/S1600536811030595/lx2194Isup3.cml

Additional supplementary materials:  crystallographic information; 3D view; checkCIF report

## Figures and Tables

**Table 1:** Hydrogen-bond geometry (Å, °)

*D*—H⋯*A*	*D*—H	H⋯*A*	*D*⋯*A*	*D*—H⋯*A*
N1—H1*A*⋯O12^i^	0.88 (2)	1.95 (3)	2.769 (5)	155 (4)
N1—H1*B*⋯O11^ii^	0.89 (2)	1.99 (3)	2.835 (5)	159 (4)
N2—H2*N*⋯O13^iii^	0.86 (2)	1.92 (2)	2.783 (4)	175 (4)
O2—H2*O*⋯O10^iii^	0.83 (2)	1.96 (2)	2.782 (4)	173 (5)
N3—H3*A*⋯O3^iv^	0.89 (2)	2.10 (2)	2.958 (5)	161 (4)
N3—H3*B*⋯O1	0.91 (2)	1.90 (2)	2.788 (5)	163 (4)
N3—H3*C*⋯O9^ii^	0.92 (2)	1.95 (2)	2.833 (5)	160 (4)
O3—H3*O*⋯O4	0.82 (2)	2.39 (5)	2.732 (4)	106 (4)
O3—H3*O*⋯O10^v^	0.82 (2)	1.96 (3)	2.720 (4)	153 (4)
N4—H4*N*⋯O2^iv^	0.87 (2)	2.01 (3)	2.811 (5)	154 (4)
O4—H4*O*⋯O9	0.84 (2)	2.02 (2)	2.854 (4)	171 (5)
O6—H6*O*⋯O12^vi^	0.82 (2)	1.81 (3)	2.598 (4)	160 (5)
O7—H7*O*⋯O6	0.84 (2)	2.15 (4)	2.667 (4)	119 (4)
O7—H7*O*⋯O4^vi^	0.84 (2)	2.31 (3)	3.085 (4)	154 (4)
O8—H8*O*⋯O11	0.83 (2)	1.99 (2)	2.794 (4)	164 (5)
O8—H8*O*⋯O13	0.83 (2)	2.34 (4)	2.892 (4)	125 (4)
O9—H9*A*⋯O12	0.85 (2)	2.14 (3)	2.875 (4)	145 (5)
O9—H9*A*⋯O1^ii^	0.85 (2)	2.51 (5)	3.036 (4)	121 (4)
O9—H9*B*⋯O5	0.83 (2)	2.03 (3)	2.794 (4)	152 (5)
C3—H3⋯O13^iii^	0.95	2.39	3.079 (5)	129
C7—H7⋯O9	0.95	2.57	3.291 (5)	133

Symmetry codes: (i) 
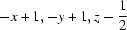
; (ii) 
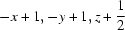
; (iii) 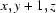; (iv) 
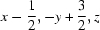
; (v) 
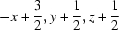
; (vi) 
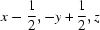
.

**Fig. 1.**
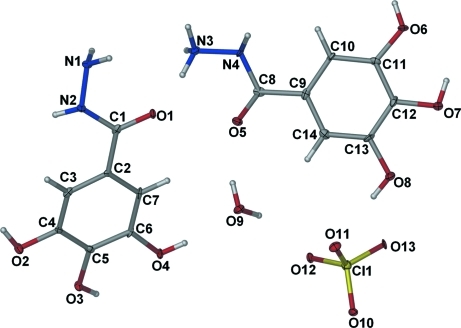
Molecular structure of the title compound with thermal ellipsoids at the 50% probability level. Hydrogen atoms are drawn as spheres of arbitrary radius.

**Fig. 2.**
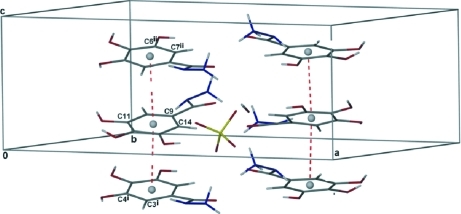
A view of the π..π interactions (dotted lines) in the crystal structure of the title compound. [Symmetry codes: (i) - x + 1, - y + 1, z - 1/2; (ii) - x + 1, - y + 1, z +1/2.]

## supplementary crystallographic information

### Comment

The title compound was obtained unexpectedly during an attempt to prepare a nickel(II) complex of 3,4,5–trihydroxybenzohydrazide. The structure contains a hydrazidium cation and a neutral hydrazide molecule. The cationic hydrazidium moiety is almost planar, within which the aromatic ring, C9—C14, and the N3/N4/C8/O5 plane make a dihedral angle of 12.5 (2)° which is smaller than the corresponding value [30.52 (3)°] in 3,4,5–trimethoxybenzohydrazidium chloride (Saeed *et al.*, 2008). The dihedral angle between the aromatic ring of the neutral hydrazide, C2—C7, and the N1/N2/C1/O1 plane is 19.0 (3)° which is larger than what was reported for 3,4,5–trimethoxybenzohydrazide hemihydrate [9.27 (10)°, Zareef *et al.*, 2006].

The crystal structure contains perchlorate anions and water molecules which are bonded to the hydrazidium and hydrazide moieties *via* O—H···O, N—H···O and C—H···O interactions (Table 1) to form a three–dimensional supramolecualr network. The crystal packing (Fig. 2) is consolidated by π–π interactions between the benzene rings of the hydrazidium and hydrazide moieties, with a *Cg*1···*Cg*2^i^ and a *Cg*1···*Cg*2^ii^ distances of 3.486 (3) and 3.559 (3) Å, respectively (*Cg*1 and *Cg*2 are the centroids of the C9–C15 benzene ring and the C2–C7 benzene ring, respectively).

### Experimental

A solution of nickel(II) perchlorate monohydrate (0.585 g, 1.6 mole) in ethanol (50 ml) was added slowly to an ethanolic solution of gallic hydrazide (0.60 g, 3.3 mmol) in the same solvent. A few drops of triethylamine was added and the mixture was refluxed for 5 h. The precipitate was filtered and recrystallized from DMSO to give the colorless crystals of the title compound.

### Refinement

The C–bound H atoms were placed at calculated positions and were treated as riding on their parent C atoms with C—H = 0.95 Å. The N– and O–bound H atoms were located in a difference Fourier map, and refined with distance restraints of O—H = 0.84 (2) Å, N2—H = 0.88 (2) Å, N1—H and N3—H = 0.91 (2) Å. For all H atoms, *U*_iso_(H) was set to 1.2(1.5 for hydroxyl)*U*_eq_(carrier atom). The displacement ellipsoids of C6 were restrained using command ISOR (0.01). The structure was a racemic twin and the twin parameter refined to 0.28 (11). An absolute structure was established using anomalous dispersion effects; 1545 Friedel pairs were not merged. The most disagreeable reflections with delta(F2)/e.s.d. > 10 were omitted (3 reflections).

### Crystal data

**Table:**

C_7_H_9_N_2_O_4_^+^·ClO_4_^−^·C_7_H_8_N_2_O_4_·H_2_O	*F*(000) = 1008
*M**_r_* = 486.78	*D*_x_ = 1.774 Mg m^−^^3^
Orthorhombic, *P**n**a*2_1_	Mo *K*α radiation, λ = 0.71073 Å
Hall symbol: P 2c -2n	Cell parameters from 1227 reflections
*a* = 20.1213 (7) Å	θ = 3.2–27.0°
*b* = 12.9178 (4) Å	µ = 0.30 mm^−^^1^
*c* = 7.0122 (2) Å	*T* = 100 K
*V* = 1822.63 (10) Å^3^	Needle, colorless
*Z* = 4	0.15 × 0.04 × 0.03 mm

### Data collection

**Table:**

Bruker APEXII CCD diffractometer	3393 independent reflections
Radiation source: fine-focus sealed tube	2653 reflections with *I* > 2σ(*I*)
graphite	*R*_int_ = 0.092
φ and ω scans	θ_max_ = 25.5°, θ_min_ = 2.6°
Absorption correction: multi-scan (*SADABS*; Sheldrick, 1996)	*h* = −24→24
*T*_min_ = 0.957, *T*_max_ = 0.991	*k* = −15→15
14383 measured reflections	*l* = −8→8

### Refinement

**Table:**

Refinement on *F*^2^	Secondary atom site location: difference Fourier map
Least-squares matrix: full	Hydrogen site location: difference Fourier map
*R*[*F*^2^ > 2σ(*F*^2^)] = 0.049	H atoms treated by a mixture of independent and constrained refinement
*wR*(*F*^2^) = 0.109	*w* = 1/[σ^2^(*F*_o_^2^) + (0.0535*P*)^2^] where *P* = (*F*_o_^2^ + 2*F*_c_^2^)/3
*S* = 1.03	(Δ/σ)_max_ < 0.001
3393 reflections	Δρ_max_ = 0.39 e Å^−^^3^
335 parameters	Δρ_min_ = −0.53 e Å^−^^3^
22 restraints	Absolute structure: Flack (1983), 1545 Friedel pairs
Primary atom site location: structure-invariant direct methods	Flack parameter: 0.28 (11)

### Special details

**Table:**

Geometry. All e.s.d.'s (except the e.s.d. in the dihedral angle between two l.s. planes) are estimated using the full covariance matrix. The cell e.s.d.'s are taken into account individually in the estimation of e.s.d.'s in distances, angles and torsion angles; correlations between e.s.d.'s in cell parameters are only used when they are defined by crystal symmetry. An approximate (isotropic) treatment of cell e.s.d.'s is used for estimating e.s.d.'s involving l.s. planes.
Refinement. Refinement of *F*^2^ against ALL reflections. The weighted *R*-factor *wR* and goodness of fit *S* are based on *F*^2^, conventional *R*-factors *R* are based on *F*, with *F* set to zero for negative *F*^2^. The threshold expression of *F*^2^ > σ(*F*^2^) is used only for calculating *R*-factors(gt) *etc*. and is not relevant to the choice of reflections for refinement. *R*-factors based on *F*^2^ are statistically about twice as large as those based on *F*, and *R*- factors based on ALL data will be even larger.

### Fractional atomic coordinates and isotropic or equivalent isotropic displacement parameters (Å^2^)

**Table:**

	*x*	*y*	*z*	*U*_iso_*/*U*_eq_	
O1	0.47204 (14)	0.7004 (2)	0.1226 (4)	0.0100 (7)	
O2	0.74400 (13)	0.8918 (2)	0.1487 (5)	0.0141 (7)	
H2O	0.722 (2)	0.945 (2)	0.133 (8)	0.021*	
O3	0.78618 (15)	0.7058 (2)	0.2858 (4)	0.0107 (7)	
H3O	0.795 (2)	0.652 (2)	0.343 (6)	0.016*	
O4	0.70017 (14)	0.5426 (2)	0.3081 (4)	0.0121 (7)	
H4O	0.6714 (18)	0.496 (3)	0.301 (7)	0.018*	
N1	0.42795 (17)	0.8931 (3)	0.1273 (5)	0.0095 (8)	
H1A	0.4015 (19)	0.856 (3)	0.054 (5)	0.011*	
H1B	0.416 (2)	0.880 (3)	0.247 (3)	0.011*	
N2	0.49692 (17)	0.8702 (3)	0.1213 (6)	0.0119 (9)	
H2N	0.5225 (18)	0.921 (2)	0.155 (7)	0.014*	
C1	0.5142 (2)	0.7687 (3)	0.1322 (6)	0.0104 (9)	
C2	0.58680 (19)	0.7498 (3)	0.1540 (7)	0.0075 (8)	
C3	0.6324 (2)	0.8282 (3)	0.1261 (6)	0.0107 (9)	
H3	0.6182	0.8929	0.0760	0.013*	
C4	0.6987 (2)	0.8133 (3)	0.1707 (6)	0.0089 (9)	
C5	0.7199 (2)	0.7175 (3)	0.2372 (6)	0.0097 (9)	
C6	0.6750 (2)	0.6371 (3)	0.2535 (6)	0.0097 (9)	
C7	0.6085 (2)	0.6524 (3)	0.2159 (6)	0.0101 (9)	
H7	0.5776	0.5974	0.2317	0.012*	
O5	0.46214 (14)	0.4747 (2)	0.3255 (4)	0.0143 (7)	
O6	0.23020 (14)	0.1848 (2)	0.2092 (4)	0.0150 (8)	
H6O	0.2019 (19)	0.218 (3)	0.268 (6)	0.022*	
O7	0.32391 (15)	0.0490 (2)	0.1104 (5)	0.0153 (7)	
H7O	0.2861 (14)	0.045 (4)	0.162 (7)	0.023*	
O8	0.44941 (15)	0.1051 (2)	0.0634 (5)	0.0140 (7)	
H8O	0.4886 (12)	0.125 (3)	0.071 (7)	0.021*	
N3	0.3796 (2)	0.6259 (3)	0.3853 (5)	0.0119 (8)	
H3A	0.3444 (16)	0.666 (3)	0.360 (6)	0.014*	
H3B	0.4158 (16)	0.643 (4)	0.314 (6)	0.014*	
H3C	0.394 (2)	0.631 (4)	0.509 (3)	0.014*	
N4	0.35607 (18)	0.5261 (3)	0.3343 (5)	0.0119 (8)	
H4N	0.3207 (15)	0.531 (4)	0.264 (6)	0.014*	
C8	0.4038 (2)	0.4525 (3)	0.2963 (6)	0.0111 (10)	
C9	0.3795 (2)	0.3506 (3)	0.2357 (6)	0.0114 (9)	
C10	0.3123 (2)	0.3228 (3)	0.2482 (6)	0.0095 (9)	
H10	0.2800	0.3717	0.2883	0.011*	
C11	0.2938 (2)	0.2221 (3)	0.2007 (6)	0.0104 (10)	
C12	0.3409 (2)	0.1510 (3)	0.1452 (7)	0.0110 (9)	
C13	0.4064 (2)	0.1793 (3)	0.1252 (6)	0.0119 (9)	
C14	0.4260 (2)	0.2797 (3)	0.1712 (6)	0.0122 (10)	
H14	0.4713	0.2994	0.1583	0.015*	
O9	0.59168 (16)	0.4012 (2)	0.2777 (5)	0.0151 (8)	
H9A	0.596 (3)	0.344 (2)	0.334 (7)	0.023*	
H9B	0.5591 (17)	0.432 (3)	0.325 (7)	0.023*	
Cl1	0.61543 (5)	0.11830 (8)	0.15644 (16)	0.0126 (2)	
O10	0.67545 (14)	0.0734 (2)	0.0667 (4)	0.0114 (7)	
O11	0.57866 (15)	0.1783 (2)	0.0090 (4)	0.0131 (7)	
O12	0.63558 (14)	0.1903 (2)	0.3124 (4)	0.0103 (7)	
O13	0.57257 (14)	0.0391 (2)	0.2378 (4)	0.0116 (7)	

### Atomic displacement parameters (Å^2^)

**Table:**

	*U*^11^	*U*^22^	*U*^33^	*U*^12^	*U*^13^	*U*^23^
O1	0.0097 (15)	0.0054 (14)	0.0148 (16)	−0.0020 (12)	−0.0002 (14)	0.0012 (13)
O2	0.0099 (15)	0.0100 (14)	0.0225 (16)	−0.0009 (13)	−0.0038 (17)	0.0047 (16)
O3	0.0085 (16)	0.0089 (15)	0.0147 (17)	−0.0007 (13)	−0.0049 (14)	0.0025 (13)
O4	0.0091 (16)	0.0054 (15)	0.0220 (16)	−0.0008 (12)	−0.0041 (14)	0.0013 (14)
N1	0.0069 (17)	0.0144 (18)	0.007 (2)	−0.0003 (15)	−0.0011 (16)	−0.0039 (17)
N2	0.0061 (18)	0.0097 (18)	0.020 (2)	0.0008 (15)	−0.0016 (17)	−0.0005 (17)
C1	0.017 (2)	0.009 (2)	0.005 (2)	0.0016 (18)	0.003 (2)	0.0009 (19)
C2	0.0069 (19)	0.0091 (19)	0.0063 (18)	0.0001 (16)	−0.001 (2)	−0.004 (2)
C3	0.016 (2)	0.010 (2)	0.007 (2)	0.0042 (17)	−0.002 (2)	−0.0006 (19)
C4	0.007 (2)	0.008 (2)	0.011 (2)	−0.0006 (17)	0.002 (2)	−0.0016 (19)
C5	0.008 (2)	0.014 (2)	0.007 (2)	−0.0008 (18)	0.0025 (19)	−0.0014 (19)
C6	0.018 (2)	0.0026 (19)	0.008 (2)	0.0024 (18)	−0.0033 (19)	0.0021 (17)
C7	0.016 (2)	0.0049 (18)	0.009 (2)	−0.0022 (18)	−0.0016 (19)	−0.0014 (17)
O5	0.0089 (17)	0.0131 (15)	0.0209 (18)	0.0001 (13)	0.0026 (14)	0.0016 (14)
O6	0.0079 (17)	0.0131 (16)	0.024 (2)	0.0022 (13)	0.0060 (14)	−0.0018 (14)
O7	0.0101 (16)	0.0121 (15)	0.024 (2)	0.0006 (13)	0.0031 (15)	−0.0022 (14)
O8	0.0095 (16)	0.0114 (16)	0.0211 (17)	0.0021 (13)	−0.0007 (14)	−0.0036 (14)
N3	0.011 (2)	0.011 (2)	0.014 (2)	0.0029 (18)	−0.0010 (18)	−0.0016 (18)
N4	0.0106 (19)	0.0060 (17)	0.019 (2)	0.0033 (16)	−0.0024 (17)	−0.0059 (17)
C8	0.012 (2)	0.018 (2)	0.003 (2)	0.0012 (19)	−0.0009 (18)	0.0013 (19)
C9	0.017 (3)	0.0084 (19)	0.0084 (19)	0.0011 (19)	−0.003 (2)	0.0002 (17)
C10	0.014 (2)	0.005 (2)	0.009 (2)	0.0044 (18)	0.003 (2)	0.0011 (18)
C11	0.009 (2)	0.016 (2)	0.006 (2)	0.0005 (19)	0.0024 (17)	0.0025 (18)
C12	0.014 (2)	0.0065 (19)	0.013 (2)	0.0022 (17)	−0.005 (2)	−0.001 (2)
C13	0.013 (2)	0.012 (2)	0.011 (2)	0.0060 (18)	−0.0003 (19)	−0.005 (2)
C14	0.013 (2)	0.016 (2)	0.008 (2)	−0.0017 (18)	0.000 (2)	0.001 (2)
O9	0.0136 (18)	0.0108 (17)	0.0209 (19)	0.0038 (14)	0.0024 (15)	0.0002 (14)
Cl1	0.0136 (5)	0.0122 (5)	0.0120 (5)	−0.0006 (5)	0.0010 (5)	0.0001 (5)
O10	0.0094 (16)	0.0089 (15)	0.0158 (15)	0.0011 (13)	0.0038 (14)	0.0029 (13)
O11	0.0144 (17)	0.0134 (16)	0.0114 (15)	0.0056 (14)	−0.0025 (14)	0.0017 (13)
O12	0.0122 (16)	0.0094 (15)	0.0095 (15)	−0.0040 (13)	0.0011 (13)	−0.0020 (13)
O13	0.0110 (16)	0.0074 (14)	0.0164 (16)	−0.0070 (13)	0.0039 (14)	−0.0035 (13)

### Geometric parameters (Å, °)

**Table:**

O1—C1	1.227 (5)	O7—H7O	0.843 (19)
O2—C4	1.372 (5)	O8—C13	1.362 (5)
O2—H2O	0.826 (19)	O8—H8O	0.830 (19)
O3—C5	1.384 (5)	N3—N4	1.420 (5)
O3—H3O	0.82 (2)	N3—H3A	0.892 (19)
O4—C6	1.376 (5)	N3—H3B	0.911 (19)
O4—H4O	0.839 (19)	N3—H3C	0.919 (19)
N1—N2	1.420 (5)	N4—C8	1.377 (6)
N1—H1A	0.880 (19)	N4—H4N	0.867 (19)
N1—H1B	0.891 (19)	C8—C9	1.467 (6)
N2—C1	1.358 (5)	C9—C14	1.385 (6)
N2—H2N	0.863 (19)	C9—C10	1.402 (6)
C1—C2	1.488 (6)	C10—C11	1.393 (6)
C2—C3	1.381 (6)	C10—H10	0.9500
C2—C7	1.401 (6)	C11—C12	1.376 (6)
C3—C4	1.384 (6)	C12—C13	1.375 (6)
C3—H3	0.9500	C13—C14	1.393 (6)
C4—C5	1.389 (6)	C14—H14	0.9500
C5—C6	1.382 (6)	O9—H9A	0.85 (2)
C6—C7	1.379 (6)	O9—H9B	0.83 (2)
C7—H7	0.9500	Cl1—O13	1.455 (3)
O5—C8	1.226 (5)	Cl1—O10	1.480 (3)
O6—C11	1.368 (5)	Cl1—O11	1.489 (3)
O6—H6O	0.82 (2)	Cl1—O12	1.492 (3)
O7—C12	1.382 (5)		
			
C4—O2—H2O	105 (3)	H3A—N3—H3B	113 (4)
C5—O3—H3O	115 (3)	N4—N3—H3C	114 (3)
C6—O4—H4O	112 (3)	H3A—N3—H3C	113 (4)
N2—N1—H1A	117 (3)	H3B—N3—H3C	104 (4)
N2—N1—H1B	104 (3)	C8—N4—N3	116.3 (4)
H1A—N1—H1B	107 (4)	C8—N4—H4N	121 (3)
C1—N2—N1	116.8 (3)	N3—N4—H4N	110 (3)
C1—N2—H2N	124 (3)	O5—C8—N4	118.3 (4)
N1—N2—H2N	115 (3)	O5—C8—C9	125.2 (4)
O1—C1—N2	120.9 (4)	N4—C8—C9	116.3 (4)
O1—C1—C2	124.5 (3)	C14—C9—C10	120.1 (4)
N2—C1—C2	114.6 (4)	C14—C9—C8	117.6 (4)
C3—C2—C7	119.8 (4)	C10—C9—C8	122.2 (4)
C3—C2—C1	121.2 (4)	C11—C10—C9	118.9 (4)
C7—C2—C1	119.0 (4)	C11—C10—H10	120.6
C2—C3—C4	120.4 (4)	C9—C10—H10	120.6
C2—C3—H3	119.8	O6—C11—C12	114.9 (4)
C4—C3—H3	119.8	O6—C11—C10	124.6 (4)
O2—C4—C3	120.8 (4)	C12—C11—C10	120.4 (4)
O2—C4—C5	119.5 (4)	C13—C12—C11	120.7 (4)
C3—C4—C5	119.8 (4)	C13—C12—O7	118.2 (4)
C6—C5—O3	121.8 (4)	C11—C12—O7	121.1 (4)
C6—C5—C4	119.8 (4)	O8—C13—C12	117.0 (4)
O3—C5—C4	118.4 (4)	O8—C13—C14	123.2 (4)
O4—C6—C7	122.5 (4)	C12—C13—C14	119.7 (4)
O4—C6—C5	116.7 (4)	C9—C14—C13	120.0 (4)
C7—C6—C5	120.8 (4)	C9—C14—H14	120.0
C6—C7—C2	119.3 (4)	C13—C14—H14	120.0
C6—C7—H7	120.3	H9A—O9—H9B	108 (5)
C2—C7—H7	120.3	O13—Cl1—O10	112.02 (17)
C11—O6—H6O	119 (3)	O13—Cl1—O11	110.14 (18)
C12—O7—H7O	102 (3)	O10—Cl1—O11	108.30 (18)
C13—O8—H8O	111 (3)	O13—Cl1—O12	108.18 (18)
N4—N3—H3A	102 (3)	O10—Cl1—O12	109.52 (18)
N4—N3—H3B	110 (3)	O11—Cl1—O12	108.63 (17)

### Hydrogen-bond geometry (Å, °)

**Table:**

*D*—H···*A*	*D*—H	H···*A*	*D*···*A*	*D*—H···*A*
N1—H1A···O12^i^	0.88 (2)	1.95 (3)	2.769 (5)	155 (4)
N1—H1B···O11^ii^	0.89 (2)	1.99 (3)	2.835 (5)	159 (4)
N2—H2N···O13^iii^	0.86 (2)	1.92 (2)	2.783 (4)	175 (4)
O2—H2O···O10^iii^	0.83 (2)	1.96 (2)	2.782 (4)	173 (5)
N3—H3A···O3^iv^	0.89 (2)	2.10 (2)	2.958 (5)	161 (4)
N3—H3B···O1	0.91 (2)	1.90 (2)	2.788 (5)	163 (4)
N3—H3C···O9^ii^	0.92 (2)	1.95 (2)	2.833 (5)	160 (4)
O3—H3O···O4	0.82 (2)	2.39 (5)	2.732 (4)	106 (4)
O3—H3O···O10^v^	0.82 (2)	1.96 (3)	2.720 (4)	153 (4)
N4—H4N···O2^iv^	0.87 (2)	2.01 (3)	2.811 (5)	154 (4)
O4—H4O···O9	0.84 (2)	2.02 (2)	2.854 (4)	171 (5)
O6—H6O···O12^vi^	0.82 (2)	1.81 (3)	2.598 (4)	160 (5)
O7—H7O···O6	0.84 (2)	2.15 (4)	2.667 (4)	119 (4)
O7—H7O···O4^vi^	0.84 (2)	2.31 (3)	3.085 (4)	154 (4)
O8—H8O···O11	0.83 (2)	1.99 (2)	2.794 (4)	164 (5)
O8—H8O···O13	0.83 (2)	2.34 (4)	2.892 (4)	125 (4)
O9—H9A···O12	0.85 (2)	2.14 (3)	2.875 (4)	145 (5)
O9—H9A···O1^ii^	0.85 (2)	2.51 (5)	3.036 (4)	121 (4)
O9—H9B···O5	0.83 (2)	2.03 (3)	2.794 (4)	152 (5)
C3—H3···O13^iii^	0.95	2.39	3.079 (5)	129.
C7—H7···O9	0.95	2.57	3.291 (5)	133.

Symmetry codes: (i) −*x*+1, −*y*+1, *z*−1/2; (ii) −*x*+1, −*y*+1, *z*+1/2; (iii) *x*, *y*+1, *z*; (iv) *x*−1/2, −*y*+3/2, *z*; (v) −*x*+3/2, *y*+1/2, *z*+1/2; (vi) *x*−1/2, −*y*+1/2, *z*.
